# Effect of Schroth Rehabilitation Exercise Program on Scoliometer Readings, Lumbar Lordosis and Calcaneal Valgus Angle in Patients with Idiopathic Scoliosis

**Published:** 2020-04

**Authors:** Jae Yong PARK, Sang Ho KIM, Jung Chul LEE, Sung Bum JU

**Affiliations:** 1. Institute of Sport Health Science, Sunmoon University, Asan, South Korea; 2. School of Global Sport Studies, Korea University, Sejong, South Korea; 3. Department of Exercise Prescription, Dongshin University, Naju, South Korea; 4. Department of Physical Education, Busan National University of Education, Busan 47503, South Korea

## Dear Editor-in-Chief

Idiopathic scoliosis progresses during puberty and rapidly worsens as it is coupled with bone growth. A regressive spinal deformity continues to develop, even after bone growth stops. At the end of the process, the resultant spinal deformity causes severe functional disorders (1). Schroth rehabilitation exercise (SRE) is a three-dimensional exercise method that corrects scoliosis through improvement in body shape and respiratory capacity by applying rotational breathing (2). Similar to a compressed ball that regains its original shape by injection of air, SRE respiration technique applies three-dimensional exercise with optimum feedback to correct scoliosis curvature and deviation (3).

The most significant difference between general corrective exercise and SRE is in the feedback that SRE offers, whereby the patient can precisely recognize his/her image in terms of postural imbalance due to scoliosis, and in response intentionally alter breathing to improve abnormal posture (2). Although some studies verified the effect of SRE on the Cobb angle, there are few reports on change in the curvature of spinal column and lower extremity alignment according to the degree of spinal deformity. This study classified teenagers diagnosed with scoliosis into 3 groups according to the Cobb angle. The effect of 12 weeks of SRE on changes in scoliometer readings, lumbar lordosis, and the calcaneal valgus angle was examined. In addition, the study considered whether SRE improves these parameters regardless of the severity of scoliosis. Changes in scoliosis severity were reviewed in each group to assess the effectiveness of the rehabilitation program in order to establish a foundation for clinical application of SRE.

This study enrolled teenagers who had been diagnosed with idiopathic scoliosis by a rehabilitation medicine specialist, based on a Cobb angle ≥10° using whole spine anteroposterior X-ray examination. The subjects were classified into groups with a Cobb angle of 10–19° (G1 group), 20–29° (G2 group), and ≥30° (G3 group). The 12-week SRE program of 3 stages consisted of recognition of stable breathing and normal joint range of motion (weeks 0–2), Normalization of sagittal alignment application (weeks 3–8), Maintenance stage (weeks 9–12). The Cobb angle is measured radiographically. To measure the scoliometer reading, An Adam’s forward bending test was performed using the Scoliometer (National Scoliosis Foundation, Watertown, MA, USA) (4).

To determine the calcaneal valgus angle, a plumb line test was performed using the Body Balance Index System (Exbody Inc., Seoul, Korea). Data were analyzed using SPSS PC for Windows (version 15.0). To identify the main effects of the SRE program, the within-group amount of change before and after the experiment was measured, and a dependent t-test was performed. The statistical significance level (α) was set at .05.

The SRE program was able to improve scoliometer readings, lumbar lordosis, and calcaneal valgus angle, regardless of the initial scoliosis angle, and within-group changes were significant (*P*<0.05). The SRE program did not apply traditional traction therapy. The SRE routines consolidate the breathing method and inactive muscles (Table 1).

**Table 1:** Change in scoliometer readings, lumbar lordosis and the calcaneal valgus angle in each group

***Group***		***G1***			***G2***			***G3***		

**Item**		**before**	**after**	**Δ**	**before**	**after**	**Δ**	**before**	**after**	**Δ**
Scoliometer reading (°)		8.40 ±0.43	6.05 ±0.73	−2.35^***^ ±0.39	12.55 ±0.83	9.40 ±0.69	−3.15^***^ ±0.54	20.35 ±1.18	14.10 ±0.95	−6.25^***^ ±0.71
Lumbar lordosis (°)		40.21 ±1.52	44.68 ±1.28	4.47^***^ ±1.02	39.30 ±1.30	43.95 ±1.16	4.65^***^ ±0.78	39.15 ±1.75	42.65 ±1.63	3.50^***^ ±0.88
calcaneal valgus angle	Left (°)	9.55 ±0.82	6.20 ±0.78	3.35^***^− ±0.66	9.50 ±0.96	6.25 ±0.88	−3.25^***^ ±0.64	10.10 ±0.57	5.40 ±0.62	−4.70^***^ ±0.79
	Right (°)	9.50 ±1.09	7.20 ±0.74	−2.30^**^ ±0.79	9.35 ±1.10	5.50 ±0.73	−3.85^***^ ±0.84	8.45 ±0.81	6.10 ±0.78	−2.35^*^ ±0.96

Idiopathic scoliosis group with Cobb angle 10–19°(G1), 20–29° (G2), ≥30° (G3) (^* ^*P*< 0.05, ^**^ *P*< 0.01, and ^***^ *P*< 0.001)

In fact, three-dimensional exercise for spinal deformity and scoliosis was applied using a rib as a lever. SRE was effective in enhancement of spinal muscular function and balance ability in adolescents with idiopathic scoliosis, but was also helpful for activities of daily living and sports activities, and is thought to have positive effects in preventing the decline of muscular strength and balance capability that may be caused by wearing a scoliosis correction device.

This study aimed to verify the positive effects of SRE on scoliometer readings, lumbar lordosis, and calcaneal valgus angle in idiopathic scoliosis patients, and to present primary measurements of desirable growth and development in affected adolescents.

The findings of this study were as follows. The SRE program induced definite changes in scoliometer readings, lumbar lordosis, and calcaneal valgus angle, regardless of the severity of scoliosis. SRE represents conservative therapy and should be recommended for idiopathic scoliosis.
